# Influenza A viruses suppress cyclooxygenase-2 expression by affecting its mRNA stability

**DOI:** 10.1038/srep27275

**Published:** 2016-06-06

**Authors:** Sabine Eva Dudek, Katja Nitzsche, Stephan Ludwig, Christina Ehrhardt

**Affiliations:** 1 Institute of Molecular Virology (IMV), Centre for Molecular Biology of Inflammation (ZMBE), Westfaelische Wilhelms-University Muenster, Von-Esmarch-Str. 56, D-48149 Muenster, Germany; 2 Cluster of Excellence Cells in Motion, University of Muenster, Muenster, Germany

## Abstract

Infection with influenza A viruses (IAV) provokes activation of cellular defence mechanisms contributing to the innate immune and inflammatory response. In this process the cyclooxygenase-2 (COX-2) plays an important role in the induction of prostaglandin-dependent inflammation. While it has been reported that COX-2 is induced upon IAV infection, in the present study we observed a down-regulation at later stages of infection suggesting a tight regulation of COX-2 by IAV. Our data indicate the pattern-recognition receptor RIG-I as mediator of the initial IAV-induced COX-2 synthesis. Nonetheless, during on-going IAV replication substantial suppression of COX-2 mRNA and protein synthesis could be detected, accompanied by a decrease in mRNA half-life. Interestingly, COX-2 mRNA stability was not only imbalanced by IAV replication but also by stimulation of cells with viral RNA. Our results reveal tristetraprolin (TTP), which is known to bind COX-2 mRNA and promote its rapid degradation, as regulator of COX-2 expression in IAV infection. During IAV replication and viral RNA accumulation TTP mRNA synthesis was induced, resulting in reduced COX-2 levels. Accordingly, the down-regulation of TTP resulted in increased COX-2 protein expression after IAV infection. These findings indicate a novel IAV-regulated cellular mechanism, contributing to the repression of host defence and therefore facilitating viral replication.

Influenza A viruses (IAV) are the causative agents of influenza, an acute respiratory disease, also known as the flu. Influenza occurs in seasonal outbreaks accompanied by symptoms such as fever, coryza, cough, prostration or malaise^1^. In addition to these annual epidemics, IAV also possess a pandemic potential, which may lead to worldwide epidemics with enormous morbidity, mortality and economic losses^2^. Pandemic outbreaks including the Spanish flu (H1N1) in 1918–1919 or the Hong Kong influenza (H3N2) in 1968^3^ as well as continuing transmissions of highly pathogenic avian influenza viruses to humans^2^ underline the need to understand the interplay between IAV and their host.

In humans, IAV primary infect epithelial cells of the upper respiratory tract. The innate immune system represents the first line of defence of the host to fight these infections. It comprises the barriers, cells and mediators that defend the host in a non-specific manner^4^. Invading viral RNA (vRNA) is sensed by pattern-recognition receptors (PRRs), such as the retinoic acid inducible gene-I (RIG-I). In consequence, various signalling pathways are initiated resulting in the production of type I interferons (IFNs), pro-inflammatory cytokines, chemokines and various mediators as part of the inflammatory response^5^. Among these, cyclooxygenases (COX) are important pro-inflammatory mediators. They are membrane bound enzymes that catalyse the synthesis of prostanoids derived from arachidonic acids^6^. The isozyme COX-2 is responsible for the formation of prostaglandins (PGs) that are involved in the inflammatory response^7^. One of the most abundant COX-2 products is prostaglandin E2 (PGE2) which functions in local vasodilatation, fever and activation of neutrophils and macrophages^8^. PGE2 is also a key mediator of immunopathology in chronic infections and cancer^9^.

While COX-1 is constitutively expressed maintaining housekeeping functions, COX-2 transcription is rapidly inducible by bacterial endotoxins (e.g. LPS), cytokines including IL-1 and TNF-α or growth factors^10^. In contrast to COX-1, COX-2 has a very short half-life and is regulated by 3′ untranslated region (3′UTR) cis-acting elements such as cAMP response element (CRE), NF-κB, AP-1 and CAAT enhancer binding protein (C/EBP)^11^. The Cys3His zinc finger tandem protein tristetraprolin (TTP) targets adenylate/uridylate-rich elements (AREs) in the 3′UTR promoting a rapid mRNA decay of COX-2^12^. TTP in turn can be phosphorylated by p38 MAPK or the p38-activated kinase MAPKAPK-2 (MAPK-activated protein kinase 2) that inactivates the destabilising function of TTP and renders COX-2 stable^13^,^14^,^15^,^16^,^17^.

To avoid cellular antiviral mechanisms, viruses have developed numerous strategies to manipulate or hijack cellular functions. Several studies showed virus-dependent regulation of COX-2 to support viral replication. For instance, Epstein-Barr virus suppresses PGE2 biosynthesis in human monocytes by impairment of the NF-κB activation^18^. The recombinant HBsAg, a surface antigen of hepatitis B virus, decreases COX-2 production and release of PGE2 by interfering with the ERK and NF-κB pathways^19^. Nevertheless, while increased production of PGE2 results in inhibited replication of certain viruses (e.g. adenovirus, parainfluanza virus and measles virus) it induces viral replication of others (e.g. CMV, VSV, BLV, HSV-1)^8^.

Here we show that in IAV-infected cells, COX-2 expression is tightly regulated. While the protein is induced at early times of infection^20^,^21^,^22^,^23^ via recognition of IAV vRNA by RIG-I, COX-2 expression is reduced again during on-going replication. Our data reveal that this is due to destabilisation of COX-2 mRNA by IAV-induced TTP, indicating that IAV not only leads to early induction of inflammatory mediators but also to its suppression later in the infection cycle.

## Results

### IAV replication is affected by COX-2 and its product PGE2

Previous studies revealed COX-2 as an important immune modulator in IAV infection^23^. Furthermore, COX-2 represents a potential target to alleviate clinical symptoms in IAV-caused diseases^24^,^25^,^26^.

**Figure 1:**
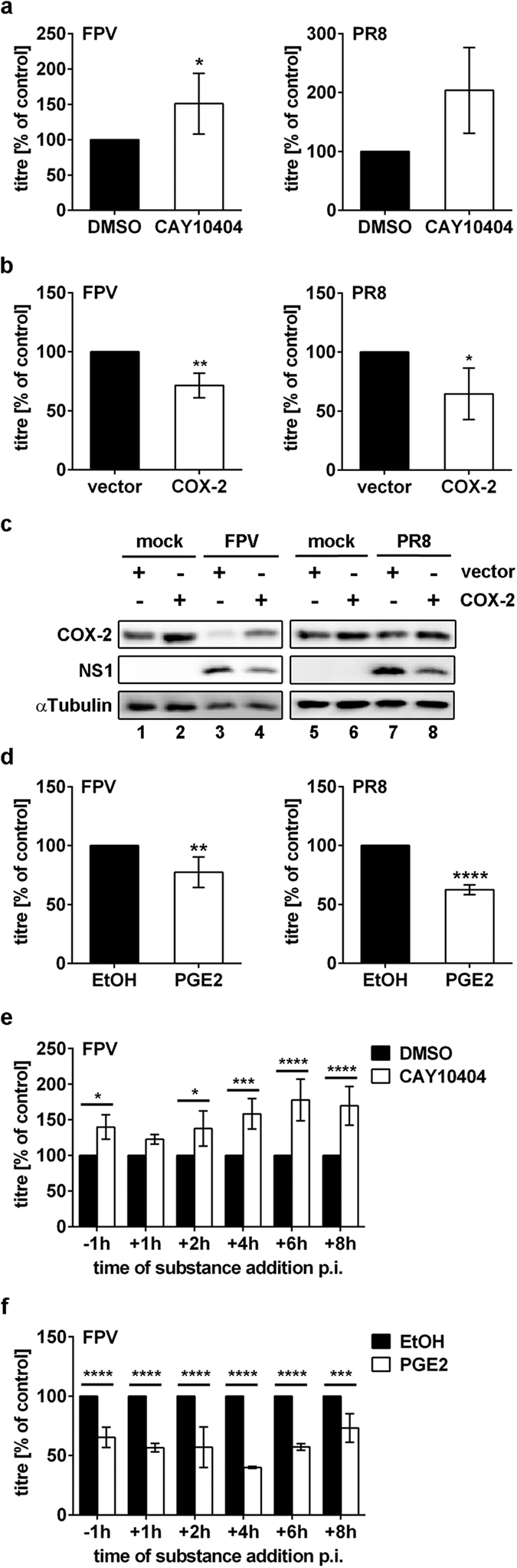
Influence of COX-2 and its product PGE2 on IAV replication. (**a,d**) A549 cells were pre-incubated either with 5 μM of CAY10404 or the solvent DMSO (**a**) or 5 μg/ml PGE2 or the solvent ethanol (EtOH) (**d**) for 1 h. Cells were infected with the IAV subtypes H7N7 (FPV; left panel) or H1N1 (PR8; right panel) at an MOI of 1 in addition to the treatment with CAY10404, PGE2 or the respective solvent for 24 h. Viral titres were determined by Standard Plaque Titration Assay. Results are depicted as mean (±s.d.) in % of solvent control (n = 4) (**a**) or (n = 6) (**d**). Statistical significance was determined by using unpaired t-test with Welch’s correction. (**a**) *P = 0.0337, and (**d**) **P = 0.0079, ****P < 0.0001. (**b,c**) A549 cells were transfected with 1 μg of *pCMV-XL6* vector encoding *cox-2* or the empty vector for 24 h. Cells were infected with 1 MOI of FPV or PR8 for 24 h. (**b**) Viral titres were determined by Standard Plaque Titration Assay as mean ( ± s.d.) in % of empty vector control (n = 5). Statistical significance was determined by using unpaired t-test with Welch’s correction. (**b**) *P = 0.0107, **P = 0.0037. (**c**) Protein lysates were subjected to WB to analyse COX-2 overexpression and viral NS1 protein expression. Shown is one representative result of n = 3. (**e,f**) A549 were either pre-incubated with 5 μM CAY10404 (**e**) or 5 μg/ml PGE2 (**f**) or the respective solvent for 1 h prior to infection or were left un-treated. Cells were infected with FPV at an MOI of 0.01. Substances were added at the indicated times of infection. Viral titres were determined by Standard Plaque Titration Assay 10 h p.i. Results are depicted as mean (±s.d.) in % of solvent control of the indicated times of infection (n = 3). Statistical significance was determined by using two-way ANOVA followed by Sidak’s test. (**e**) −1 h *P = 0.0299, + 2 h *P = 0.0433, ***P = 0.0008, ****P < 0.0001, and (**f**) ***P = 0.0003, ****P < 0.0001.

In a first set of experiments we investigated the impact of COX-2 on IAV replication. A549 human lung epithelial cells were infected with the avian influenza virus A/FPV/Bratislava/79 (H7N7) (FPV) or human isolate A/Puerto Rico/8/34 (H1N1) (PR8). Inhibition of COX-2 by the COX-2-specific inhibitor CAY10404 resulted in considerably increased viral titres 24 h post infection (p.i.) (Fig. 1a). Accordingly, the overexpression of COX-2 protein resulted in decreased viral replication 24 h p.i. Although the target cells were only partially transfected and thus the resulting titres are diluted by un-transfected background, the titre reduction was significant (Fig. 1b; FPV, P = 0.0037 and PR8, P = 0.0107). Moreover, the antiviral effect could also be verified on the level of viral protein expression, as the synthesis of the non-structural protein 1 (NS1) was also reduced (Fig. 1c). Comparable results were obtained upon stimulation of cells with the COX-2 product PGE2 (Fig. 1d; FPV, P = 0.0079 and PR8, P < 0.0001). To verify our results in non-immortalised primary cells we analysed viral titres in primary human bronchial epithelial cells (HBEpC) after CAY10404 and PGE2 treatment, confirming the inhibitory effect of COX-2 activity on IAV replication (supplementary Fig. S1a,b). To analyse the influence of COX-2 and PGE2 during on-going IAV replication A549 cells were infected up to 32 h p.i. with low doses of IAV. Multi-cycle analysis verified a substantial increase of viral titres after COX-2 inhibition (supplementary Fig. S1c). Accordingly, PGE2 stimulation resulted in decreased viral titres 32 h p.i. (supplementary Fig. S1d). Furthermore, in time-of-addition kinetics CAY10404 or PGE2 were added at different times of infection within the first replication cycle. Interestingly, a maximum increase in viral titres was observed when COX-2 was inhibited at 6 h p.i. compared to other times of CAY10404 addition (Fig. 1e +6 h P < 0.0001), while a maximum decrease in viral titres was visible upon PGE2 treatment at 4 h p.i. compared to other times of PGE2 addition (Fig. 1f).

Taken together, these results clearly indicate that COX-2, and in particular its product PGE2, plays an important role as part of the cell intrinsic innate defence against IAV.

### COX-2 expression is induced at the early stages of IAV infection

**Figure 2:**
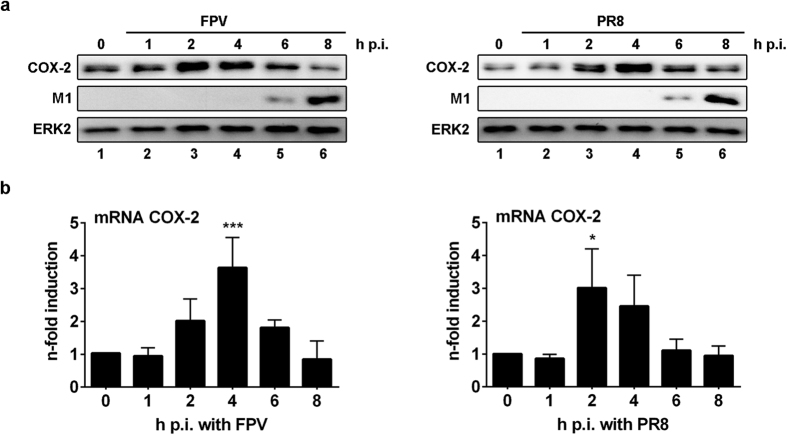
Induction of COX-2 expression after IAV infection. (**a,b**) A549 cells were infected with 5 MOI of the IAV subtypes FPV (left panel) and PR8 (right panel) for the indicated times post infection (h p.i.). (**a**) Total cellular protein extracts were subjected to WB. One representative result of n = 3 is shown. (**b**) Cellular RNA from infected cells was extracted, reverse transcribed and analysed by quantitative real-time PCR (qRT-PCR). Expressional changes of COX-2 mRNA were normalised to 0 h as n-fold induction. Results are depicted as mean n-fold (±s.d.) of n = 3. Statistical significance was determined using one-way ANOVA followed by a Dunnett’s test. *P = 0.0102, ***P = 0.0003.

To characterise molecular regulatory mechanisms of COX-2 during IAV infection in detail, we determined if its expression is induced in presence of IAV. Thus, we investigated COX-2 induction after infection of A549 cells with the IAV strains FPV or PR8 on the levels of protein expression (Fig. 2a) and mRNA synthesis (Fig. 2b). We observed highest COX-2 mRNA synthesis and COX-2 protein expression between 2 to 4 h p.i. Interestingly, thereafter, both mRNA and protein levels decreased again to reach basal levels at 8 h p.i. These results indicate that IAVs induce COX-2 expression in a well time-controlled manner.

Therefore we aimed to identify the viral stimulus and cellular sensor being responsible for COX-2 induction at early times of infection as well as the regulatory mechanism causing COX-2 decay at later stages of the IAV replication cycle.

### RIG-I mediates COX-2 induction

**Figure 3:**
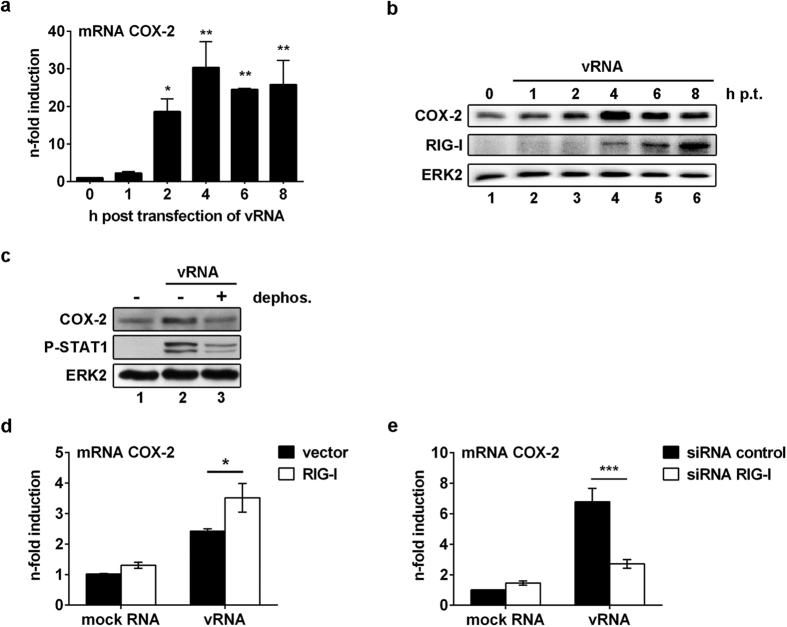
Induction of COX-2 expression after stimulation with vRNA. (**a,b**) A549 cells were transfected with 1 μg RNA, extracted from IAV-infected A549 cells (vRNA), for indicated times post transfection (h p.t.). (**a**) Cellular RNA from transfected cells was extracted, reverse transcribed and analysed by qRT-PCR. Expressional changes of COX-2 mRNA were normalised to 0 h p.t. Results are depicted as mean n-fold (±s.d.) of n = 3 independent experiments. Statistical significance was determined by using one-way ANOVA followed by a Dunnett’s test. (**a**) 2 h, P = 0.0188, 4 h, P = 0.0014, 6 h, P = 0.0047 and 8 h, P = 0.0035. (**b**) Total cellular protein extracts were analysed by WB. (**c**) 1 μg vRNA was dephosphorylated by phosphatase treatment and transfected into A549 cells for 6 h. Mock RNA and vRNA without phosphatase treatment were used as controls. Total cellular protein extracts were analysed by WB. (**d**) A549 cells were transfected with 1 μg of *pCAGGS* vector encoding for *rig-i* or empty vector as control for 24 h. Then cells were transfected with 1 μg vRNA or mock RNA for 4 h and COX-2 mRNA expression was determined via qRT-PCR as n-fold induction of empty vector mock RNA control. Results represent mean (±s.d.) of n = 3. (**e**) COX-2 mRNA expression after siRNA-mediated knock-down of RIG-I (48 h p.t.) and subsequent transfection of 1 μg vRNA or mock RNA for 4 h was determined via qRT-PCR. A negative siRNA served as control (siRNA control). Results are normalised to mock RNA control as mean (±s.d.) of n = 3 independent experiments. Statistical significance was determined by using two-way ANOVA followed by a Sidak’s test (**d,e**). (**d**) *P = 0.0264 and (**e**) **P = 0.0013.

Since the induction of COX-2 expression was observed prior to the onset of viral protein synthesis, we investigated whether early accumulation of vRNA might be the stimulus for COX-2 expression. Transfection of RNA derived from IAV-infected A549 cells (MOI = 5, 5 h p.i.) (vRNA) resulted in the induction of COX-2 already 2 h post transfection, as visible on mRNA and protein level (Fig. 3a,b). Interestingly, up-regulation of RIG-I expression as positive feedback loop to vRNA stimulation in an IFNβ-dependent manner occurred at later times p.i., when COX-2 expression decreases again (Fig. 3b). While transfection of vRNA increased COX-2 protein expression substantially, dephosphorylated vRNA failed as inducer, indicating that 5′-triphosphate RNA from IAV serves as a stimulus (Fig. 3c). Efficient de-phosphorylation of 5′-triphosphate RNA was monitored by detection of phosphorylated STAT1 (Fig. 3c).

We now aimed to identify the receptor, which is responsible for sensing these pathogen RNA structures up-stream of COX-2. The pathogen pattern-recognition receptor RIG-I is known to bind 5′-triphosphate viral RNA^27^,^28^ and also has been reported to play a role in COX-2 expression^27^. Thus, we evaluated the RIG-I-dependent regulation of COX-2. Overexpression of RIG-I provoked significant higher amounts of COX-2 mRNA after transfection of vRNA (Fig. 3d; P = 0.0264) confirming earlier findings^27^. Conversely, siRNA-mediated knock-down of RIG-I resulted in a significant reduction of vRNA-induced COX-2 mRNA synthesis (Fig. 3e; P = 0.0006). These results clearly indicate RIG-I as an essential mediator for IAV-induced COX-2 expression.

### COX-2 expression is reduced during later stages of IAV infection

**Figure 4:**
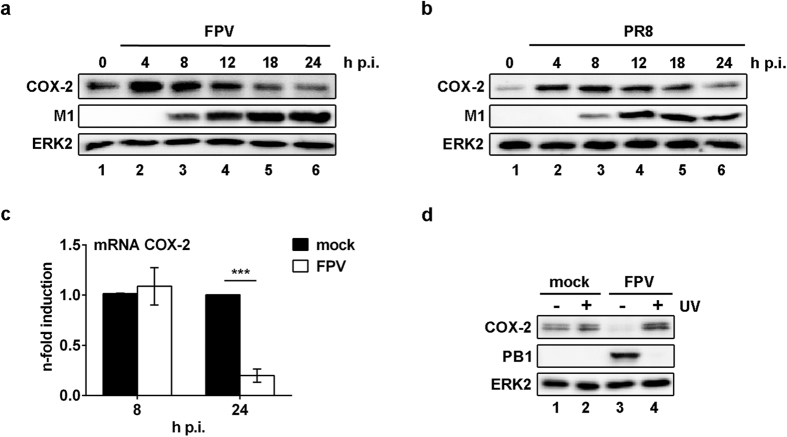
COX-2 expression is reduced during on-going IAV infection. (**a,b**) A549 cells were infected with 1 MOI of the IAV subtypes FPV (**a**) and PR8 (**b**). Total cellular protein extracts were produced 0–24 h p.i. and analysed by WB. (**c**) COX-2 mRNA synthesis in A549 cells was determined 8 h and 24 h p.i. with 1 MOI FPV by qRT-PCR. The results are normalised to the mock control and are depicted as mean (±s.d.) of n = 3 independent experiments. Statistical significance was determined by using two-way ANOVA followed by Sidak’s test. ***P = 0.0009. (**d**) Media, containing IAV virions of the subtype FPV, were treated with UV light to inactivate the virus. A549 cells were infected with UV-inactivated FPV or replicating virus for 18 h. Un-infected cells were used as control. Total cellular protein extracts were analysed by WB.

While COX-2 is rapidly induced by IAV via the vRNA-sensing RIG-I pathway, accumulation of the protein peaks around 4 h p.i. in a single-cycle experiment and declines again thereafter (Fig. 2a). To obtain closer insights into the IAV-mediated regulation of COX-2 during on-going replication, we infected A549 cells with the IAV strains FPV and PR8 for multi-cycle replication kinetics, up to 24 hours. While the expression peak was shifted to later time points due to the overlapping viral replication cycles, COX-2 amounts were considerably reduced at later stages post infection, even below the basal levels (Fig. 4a,b). This general suppression of COX-2 expression during on-going replication could also be confirmed in primary HBEpC (supplementary Fig. S2) and occurred on mRNA level, as evidenced by reduced amounts of COX-2 mRNA 24 h post FPV infection in A549 cells (Fig. 4c). Furthermore, the suppressive action on COX-2 mRNA requires productive virus replication since stimulation with UV-inactivated FPV showed no effect (Fig. 4d).

The non-structural protein NS1 of IAV is a prime viral modulator of cellular responses and has been shown to be a major determinant of virulence that affects influenza pathogenesis^28^. Thus, we analysed a possible impact of NS1 on COX-2 reduction. We infected A549 cells with the PR8 ΔNS1 mutant virus, which is unable to express NS1^29^. Interestingly, ΔNS1 even provoked higher reduction of COX-2 protein expression than the wild-type virus (supplementary Fig. S3a). As ΔNS1 virus is known to be a strong type I IFN-inducer^30^, we analysed the effect of IFNβ, which is also induced upon wild-type IAV infection via RIG-I^31^, on COX-2 expression. Stimulation with IFNβ in presence or absence of an IFNβ-neutralising antibody did not change COX-2 protein amounts substantially (supplementary Fig. S3b). Similarly, in an IFNα/β receptor knock-down context (IFNAR2) neither vRNA nor IFNβ stimulation could remarkably change COX-2 mRNA synthesis (supplementary Fig. S3c,d). This shows that neither IFNβ itself nor any IFNβ-stimulated gene (ISG) is responsible for the reduction of COX-2.

In summary, infection with replicating IAV results in reduced COX-2 amounts, which occurs independent of IFNβ or IFNβ-dependent factors.

### COX-2 decay is accompanied with loss of mRNA stability

**Figure 5:**
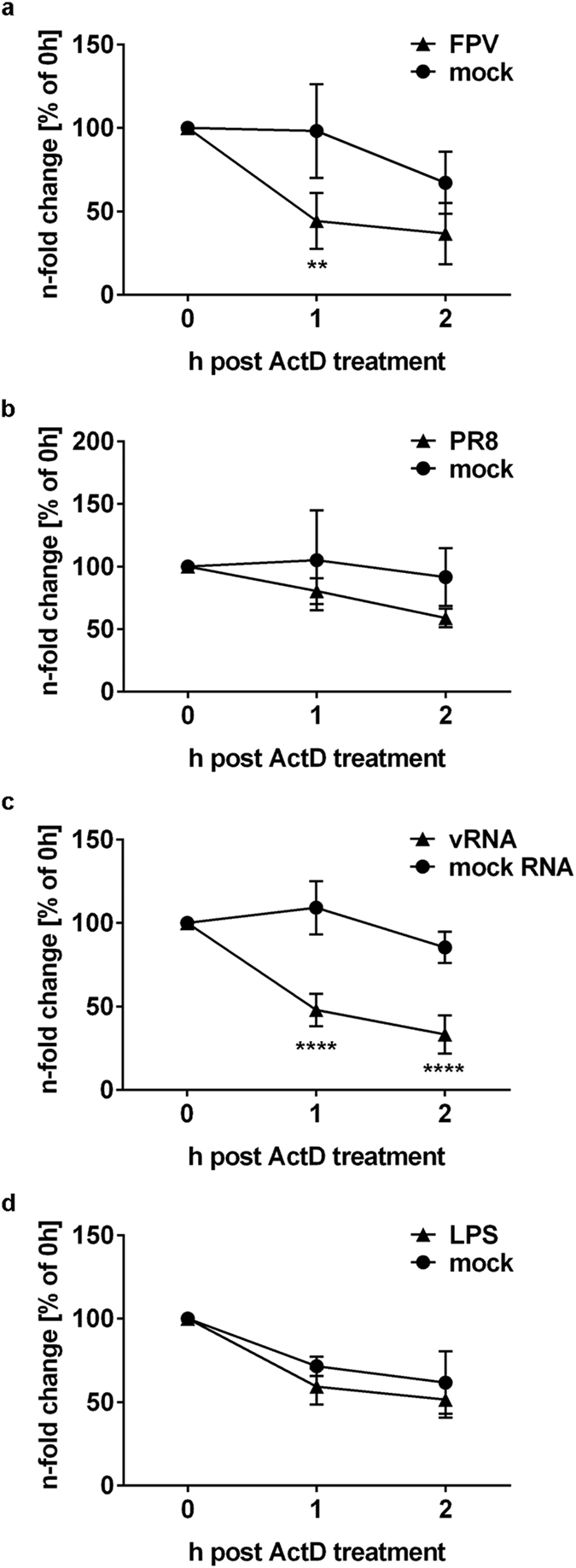
Loss of COX-2 mRNA stability. (**a–d**) COX-2 mRNA stability was analysed by inhibition of mRNA synthesis with actinomycin D (ActD). A549 cells were infected with 5 MOI of the IAV subtypes FPV (**a**) and PR8 (**b**) or transfected with 1 μg RNA extracted from IAV-infected (vRNA) or un-infected (mock RNA) A549 cells (**c**) or treated with 1 μg LPS (**d**) for 4 h. Subsequently, cells were treated with 3 μg ActD for 1 h or 2 h. After the times indicated post ActD treatment total RNA was isolated and subjected to qRT-PCR analysis for COX-2 mRNA. The n-fold induction of different treatments (0 h) was arbitrarily set to 100% and the other results were normalised to the respective 0 h time point. Data represent the mean (±s.d.) of n = 3 independent experiments. Statistical significance was determined using two-way ANOVA followed by Sidak’s test. **P = 0.0068, ****P < 0.0001.

Based on the observation that IAV infection results in reduction of COX-2 mRNA amounts in A549 cells (Fig. 4c), we analysed COX-2 mRNA half-life. Therefore, cells were treated with actinomycin D (ActD) that binds DNA at the transcription initiation complex and inhibits the formation of novel mRNA^32^. After infection of A549 cells and subsequent stimulation with ActD, RNA was isolated at different times and analysed by qRT-PCR. Infection of cells with FPV (Fig. 5a; P = 0.0068) or PR8 (Fig. 5b), as well as transfection with vRNA (Fig. 5c; P < 0.0001) caused a significantly enhanced decay of COX-2 mRNA, indicative of decreased mRNA stability. As a control we stimulated A549 cells with lipopolysaccharide (LPS), a bacterial component that is a well-known inducer of COX-2 expression^33^ prior to ActD stimulation. Indeed, LPS treatment did not cause any remarkable differences in COX-2 mRNA stability (Fig. 5d). Thus, our results indicate an IAV-mediated effect on COX-2 mRNA stability.

### IAV mediates reduction of COX-2 via TTP

**Figure 6:**
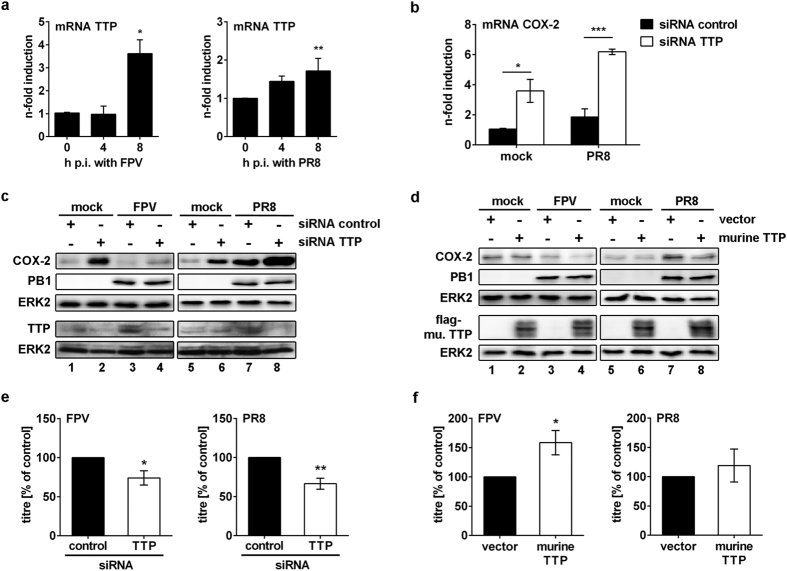
IAV-mediated reduction of COX-2 expression via TTP. (**a**) A549 cells were infected with 5 MOI of the IAV subtypes FPV (left panel) or PR8 (right panel). After indicated times p.i. RNA from cell lysates was isolated and reverse transcribed. TTP mRNA synthesis was analysed by qRT-PCR. The results were normalised to the time point 0 h and are depicted as mean n-fold (±s.d.) of n = 3 independent experiments. Statistical significance was determined by using one-way ANOVA followed by Dunnett’s test. FPV, *P = 0.0129, PR8, **P = 0.0091. (**b,c,e**) For knock-down of TTP A549 cells were transfected with 10 μM TTP siRNA or a negative control (siRNA control). After 48 h the transfected cells were infected with 5 MOI of FPV (**c**) or 5 MOI of PR8 (**b,c**), or 1 MOI of FPV or PR8 (**e**). (**b**) 8 h p.i. COX-2 mRNA synthesis was analysed by qRT-PCR. The results were normalised to mock-infected control and are depicted as mean n-fold (±s.d.) of n = 3 independent experiments. Statistical significance was determined using two-way ANOVA followed by Sidak’s test. *P = 0.0100, ***P = 0.0004. (**c**) 8 h p.i. total cellular protein extracts were analysed by WB. (**d,f**) A549 cells were transfected with 1 μg of *p3XFLAG-CMV-7.1* vector encoding murine *ttp* or the empty vector as control for 24 h. Subsequently, cells were infected with 5 MOI of FPV or PR8 (**d**), or 1 MOI of FPV or PR8 (**f**). (**d**) Total cellular protein extracts were analysed by WB 8 h p.i. (**e,f**) Viral titres were determined 24 h p.i. by Standard Plaque Titration Assay. Results are depicted as mean (±s.d.) in % of siRNA control (**e**) or empty vector control (**f**) (n = 3). Statistical significance was determined by using unpaired t-test with Welch’s correction. (**e**) FPV, *P = 0.0397, and PR8, **P = 0.0024, and (**f**) FPV, *P = 0.0111.

COX-2 mRNA stability is regulated by specific RNA-binding proteins such as human antigen R (HuR) or tristetraprolin (TTP). Since TTP was shown to destabilise several ARE-containing mRNAs including COX-2^15^, we further analysed the role of TTP during IAV-regulated COX-2 suppression. Infection with FPV or PR8 resulted in significantly increased TTP mRNA synthesis 8 h p.i. (Fig. 6a; FPV, P = 0.0129; PR8, P = 0.0091). In consequence, we analysed the effect of TTP on COX-2 mRNA expression. We observed an enhancement in COX-2 mRNA expression upon TTP knock-down that was further increased upon IAV infection (Fig. 6b). Similarly, the knock-down of TTP (Fig. 6c lanes 2 and 6) as well as additional IAV infection (Fig. 6c lanes 4 and 8) resulted in increased COX-2 protein accumulation. Corresponding to the changes in TTP mRNA synthesis, IAV infection resulted in enhanced TTP protein expression (Fig. 6c lanes 3 and 7). Overexpression of murine TTP confirmed its destabilising function and led to the down-regulation of COX-2 protein expression in A549 cells after IAV infection (Fig. 6d lanes 4 and 8).

In accordance to our previous results on viral titres after COX-2 modulation we analysed the effect of TTP on IAV replication. While IAV titres were significantly decreased upon TTP knock-down (Fig. 6e FPV, P = 0.0397, and PR8, P = 0.0024) overexpression of murine TTP resulted in increased viral titres (Fig. 6f).

**Figure 7:**
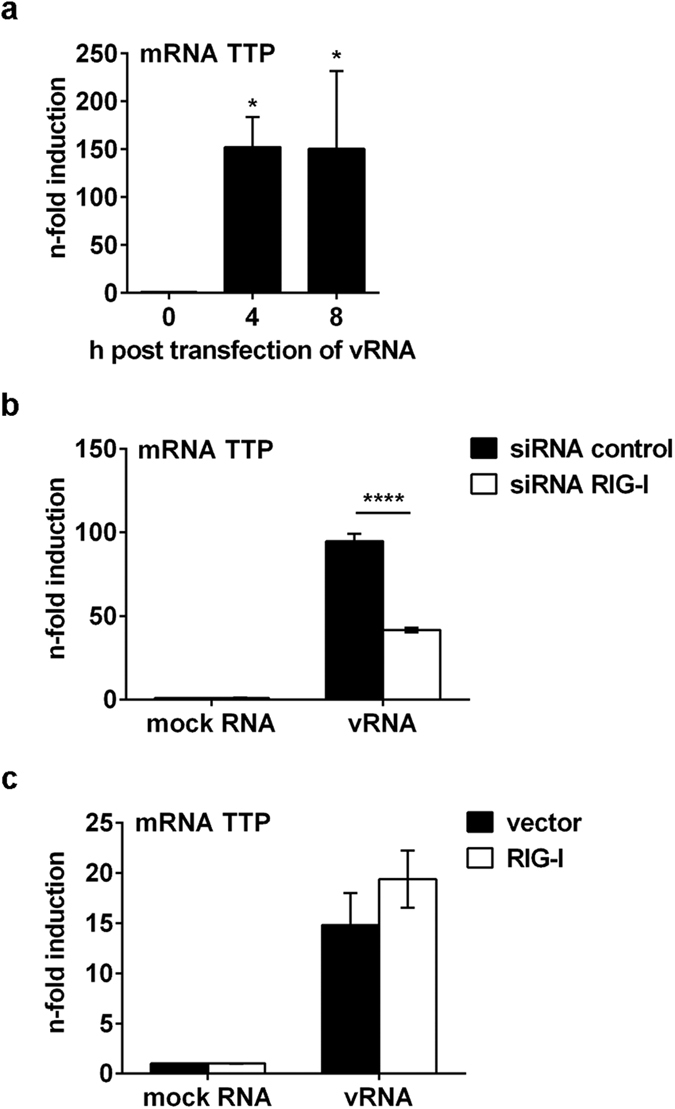
TTP mRNA induction via detection of IAV RNA by RIG-I. (**a**) A549 cells were transfected with 1 μg RNA isolated from IAV-infected A549 cells (vRNA) for indicated times. RNA was isolated, reverse transcribed and subjected to qRT-PCR analysis for TTP mRNA. Results were normalised to 0 h time point as mean n-fold (±s.d.) of n = 3 independent experiments. Statistical significance was determined using one-way ANOVA followed by Dunnett’s test. 4 h, *P = 0.0180 and 8 h, *P = 0.0191. (**b,c**) A549 cells were either transfected with an siRNA specific for RIG-I or a negative control siRNA (siRNA control) for 48 h (**b**) or with 1 μg of *pCAGGS* vector encoding for *rig-i* or empty vector as control for 24 h (**c**). Subsequently, cells were transfected with 1 μg vRNA or mock RNA for 4 h and TTP mRNA expression was determined via qRT-PCR as n-fold induction of negative siRNA mock RNA control (**b**) or empty vector mock RNA control (**c**). Results represent mean (±s.d.) of n = 3. Statistical significance was determined using two-way ANOVA followed by Sidak’s test. ****P < 0.0001.

As vRNA also destabilises COX-2 mRNA (Fig. 5c) we analysed whether vRNA is sufficient to induce TTP. Indeed, transfection of vRNA resulted in significantly induced TTP mRNA expression (Fig. 7a; 4 h, P = 0.0180 and 8 h, P = 0.0191). Remarkably, this induction also depends on the detection of vRNA via the pattern-recognition receptor RIG-I as seen in a significant loss of TTP mRNA induction in RIG-I knock-down cells (P < 0.0001) (Fig. 7b) and an enhanced induction of TTP mRNA after RIG-I overexpression (Fig. 7c). Furthermore, the induction of TTP mRNA was independent of type I IFN signalling as stimulation with different amounts of IFNβ (supplementary Fig. S4a) as well as stimulation with vRNA in IFNAR2 knock-down cells (supplementary Fig. S4b) did not show differences to the corresponding controls.

Taken together, our findings clearly demonstrate an IAV-mediated inhibition of COX-2 expression, controlled by the IAV-induced cellular product TTP, an mRNA destabilising protein.

## Discussion

It has been shown that viral infections stimulate the expression of a number of pro-inflammatory genes, such as COX-2. Interestingly, the COX-2 product PGE2 participates in the regulation of viral replication and the modulation of pro-inflammatory responses to infections^8^. IAV is reported to induce the expression of the pro-inflammatory mediator COX-2 during infection of human bronchial epithelial cells^23^, as well as in peripheral blood mononuclear cells (PBMCs) of infected patients^21^. In the present study we identified viral RNA as responsible factor for the immediate early induction of COX-2 after IAV infection in human lung epithelial cells. Furthermore, we showed that IAV suppresses COX-2 expression at later stages of infection accompanied by a considerable loss of mRNA stability in A549 cells.

COX-2 activation leads to the release of prostaglandins, such as PGE2 that are involved in inflammatory response and regulation of viral replication^7^,^8^. Our data clearly demonstrate an antiviral potential of COX-2 and its product PGE2 on IAV replication in the lung epithelial cell line A549 (Fig. 1) as well as in primary lung epithelial cells (supplementary Fig. S1). Since non-steroidal anti-inflammatory drugs (NSAIDs) and in particular specific COX-2 inhibitors (COXIBs) increasingly acquired relevance as therapeutic agents, we investigated their effects on IAV replication. Our results suggest that inhibition of the antiviral factor COX-2 by anti-inflammatory drugs, such as the COX-2-specific inhibitor CAY10404 that was used in our study, may have detrimental outcome for treatment during an influenza disease because of a likely enhancement of viral replication. So far, the effects of COX-2-induced responses during IAV infection are discussed controversial. Recently, we have shown that the usage of aspirin, a NSAID inhibiting both COX-1 and COX-2, in context of IAV infections led to a reduced viral replication in A549 cells as well as in mice^34^. Nonetheless, the aspirin-mediated antiviral effect is primarily due to inhibition of NF-κB-induced expression of pro-apoptotic factors supporting the export of viral ribonucleoproteins out of the nucleus^34^. In fact it seems that this effect is predominant over the COX-inhibiting effects, because the COX-1/COX-2 inhibitor indometacin did not show any antiviral effects in effective concentrations in the setting of these studies^34^. Other studies dealing with novel COX-2 inhibitors or other PGE2 synthesis inhibitors indicated anti-influenza activities of these substances, leading to reduced pathology in mice^26^,^35^, while the administration of PGE2 reversed this phenotype^36^. Experiments with genetically deficient mice might be helpful here and should be taken as the gold standard because of the lack of off-target effects that might occur with inhibitors. These studies clearly showed that viral titres increased in COX-2^−/−^ mice but not in COX-1^−/−^ mice, while PGE2 levels were similar in COX-2^−/−^ mice compared to wild-type mice^37^,^38^. In summary, while there is a clear tendency to conclude that COX-2 inhibition or genetic deletion leads to enhanced virus titres, the complex interplay of the different COX isozymes and their diverse down-stream products fulfilling different functions during antiviral immunity may hamper definite conclusions to date^35^,^38^.

When we analysed effects of COX-2 activity on different steps during the IAV life cycle in more detail by a time-of-addition kinetics, we observed a time-regulated antiviral effect of the COX-2 product PGE2. The highest reduction in IAV titres was achieved upon addition of PGE2 4 h p.i. (Fig. 1f), correlating with highest levels in COX-2 mRNA synthesis and protein expression as well as the onset of viral protein synthesis (Fig. 2). Accordingly, PGE2 might interfere with these viral processes in lung epithelial cells. However, these observations also raise the question if the regulatory mechanisms of COX-2 activity might strongly be controlled in a time-dependent manner during viral infection.

Indeed, at early stages of IAV infection we detected an increase in COX-2 protein and mRNA expression with a peak at 4 h p.i. (Fig. 2). These results go along with the observations made for other viruses such as the hepatitis B virus or Epstein-Barr virus inducing COX-2 expression^39^,^40^. Similar induction levels have been observed when A549 cells were exposed to vRNA. However, dephosphorylated vRNA failed as inducer, indicating that 5′-triphosphate RNA from IAV serves as a stimulus for COX-2 induction (Fig. 3a,c).

The first line of non-specific immune response is generated by sensing IAV vRNA via PRRs, such as RIG-I^41^,^42^. RIG-I-mediated signalling results in type I IFN release with subsequent transcription of antiviral acting genes, immune cell activation and stimulation of the adaptive immune system; however, it also encourages cells to express COX-2^43^,^44^. IFN itself triggers the expression of IFN-inducing proteins, such as RIG-I, via a positive feedback loop^45^,^46^. Moreover, RIG-I has been reported to play a role in the expression of COX-2 at the transcriptional level in endothelial cells^47^. Indeed, our data verified RIG-I as the mediator for the early IAV-induced COX-2 expression upon recognition of vRNA in A549 cells (Fig. 3d,e).

At on-going infection, however, a significant decay of COX-2 has been detected (Fig. 4). Since inactivated IAV lost its suppressive impact, we concluded that viral factors or cellular products that are induced by replicating IAV mediate the reduction of COX-2 levels. In this context we could exclude that accumulation of the major viral cell-modulator NS1, a prominent signalling suppressor^29^, acts as causal agent for reduced COX-2 expression. Several studies described different types of interferons (IFNs) to be involved in the regulation of COX-2 expression. While IFNα was shown to induce COX-2 expression in A549 cells via STAT1 activation^48^,^49^, IFNγ and IFNβ inhibited COX-2 expression in different cell types by blocking its promoter activity in a STAT1-independent manner^50^,^51^. As type I IFNs, and in particular IFNβ is one of the best characterised down-stream targets of RIG-I being induced by IAV vRNA^31^,^52^, we analysed whether our observations might depend on IFNβ. However, we were able to exclude that IFNβ or an IFNβ-dependent factor is responsible for the reduced COX-2 mRNA accumulation (supplementary Fig. S3).

Modulation of COX-2 synthesis is a complex process regulated at many levels. Multiple signalling pathways depending on cell type and stimulus lead to activation of COX-2 transcription^53^. Furthermore, COX-2 mRNA stabilisation is regulated by specific mRNA-binding proteins and posttranslational degradation limits the COX-2-dependent immune response^11^,^54^,^55^. Reduction of COX-2 mRNA accumulation after infection indicates an effect on COX-2 transcription or at a posttranscriptional level. Indeed, we found that COX-2 mRNA half-life was altered in IAV-infected A549 cells. Our results showed a considerable decay of mRNA stability after 4 h of infection with IAV as well as after transfection with vRNA while stimulation with LPS, the prototypical bacterial pathogen-associated molecular pattern, was unable to destabilise COX-2 mRNA (Fig. 5). These findings clearly indicate an IAV-mediated effect on regulation of COX-2 at the posttranscriptional level. These observations suggest that signalling cascades following detection of vRNA by RIG-I play an important role in the regulation of COX-2. Moreover, COX-2 mRNA stability is regulated at the 3′ untranslated region (3′UTR) that contains multiple AU-rich elements (ARE). Interactions at this region regulate the stability of COX-2 mRNA^11^. Since the RNA-binding protein tristetraprolin (TTP) is known to destabilise several ARE-containing mRNAs including COX-2^13^, we further examined a particular role of TTP during IAV infections. As described in previous studies, high levels of TTP decrease COX-2 expression (Fig. 6)^56^. As expected, TTP knock-down led to enhanced accumulation of COX-2 protein (Fig. 6). Nonetheless, infection with IAV resulted in enhanced TTP protein and mRNA expression suggesting an IAV-dependent COX-2 suppression mediated by TTP induction. Remarkably, the effects of TTP manipulation observed on viral titres (Fig. 6e,f) were in line with the results of COX-2 manipulation on IAV replication (Fig. 1a,b). The knock-down of TTP raised the COX-2 mRNA stability and resulted in enhanced COX-2 protein expression and activity. Concomitantly, decreased viral titres were observed upon TTP knock-down (Fig. 6e) as well as the overexpression of COX-2 (Fig. 1b), while vice versa effects were monitored when TTP was overexpressed or COX-2 inhibited (Figs 1a and 6f).

Interestingly, previous studies established a link between interferon responses and TTP-mediated mRNA degradation. IFN is reported to induce TTP via p38 MAPK and STAT1^57^. In addition, activation of p38 MAPK results in the phosphorylation of TTP that inactivates its destabilising function^16^,^58^. Although, our data indicate that TTP mRNA-induction is independent of type I IFN-mediated signalling (supplementary Fig. S4) we were able to unravel a RIG-I-mediated induction of TTP mRNA synthesis upon induction of the vRNA sensing pathway (Fig. 7). Remarkably, IAV infection leads to induction of several MAPK including p38 MAPK (reviewed in^59^) and activity of this kinase is needed for IAV-induced PGE2 production^23^. Our data perfectly fit into this context as they close the gap of IAV-mediated regulatory mechanism of COX-2 induction and PGE2 production.

**Figure 8:**
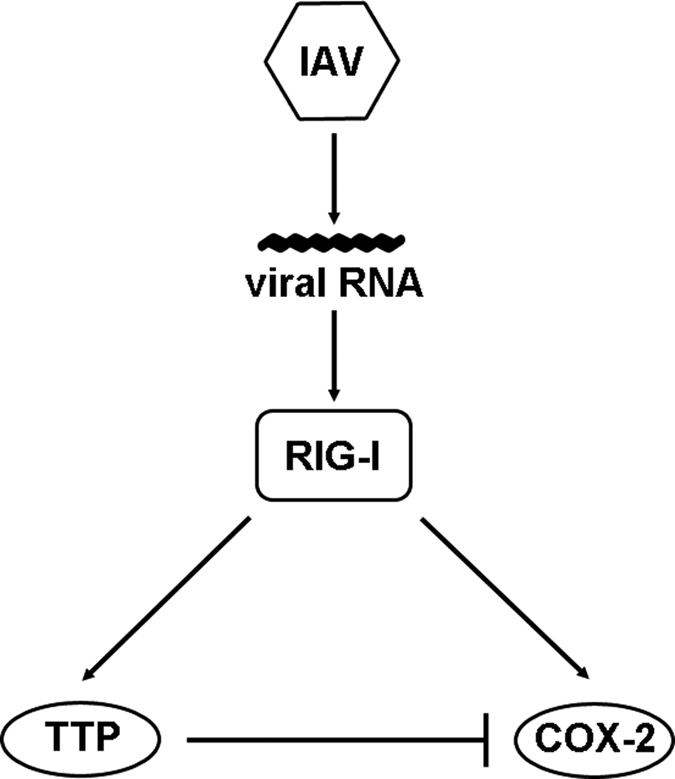
Model for the IAV-mediated down-regulation of COX-2. IAV-induced activation of RIG-I initiates various signalling pathways resulting in the production of mediators as part of the inflammatory response including COX-2. In addition, IAV induces TTP via activation of RIG-I and mediates an inhibitory mechanism in which TTP promotes COX-2 mRNA degradation.

In summary, we unravelled a novel strategy of IAVs to evade the inflammatory response in human airway epithelial cells. Our data point to a feedback mechanism that induces the TTP-mediated degradation of COX-2 upon accumulation of vRNA and recognition by RIG-I (Fig. 8). Here, for the first time it has been shown that IAV induces an inhibitory regulating mechanism to arrest an inflammatory response and hijack the cellular immune response to ensure its sufficient viral replication.

## Methods

### Cell lines, viruses and UV-inactivation

Madin Darby canine kidney cells (MDCK) were grown in minimal-essential medium (MEM, PAA Laboratories) while the human alveolar epithelial cells (A549) were cultured in Dulbecco’s modified Eagle’s medium (DMEM, PAA Laboratories), both complemented with 10% foetal bovine serum (FBS, Biochrom). The cell lines were maintained in a humidified atmosphere at 37°C with 5% CO_2_.

The avian influenza virus A/FPV/Bratislava/79 (H7N7, FPV) and the human influenza virus A/Puerto Rico/8/34 (H1N1, PR8) were obtained from the virus strain collection of the Institute of Virology, Giessen (Germany) and propagated and passaged in MDCK cells.

To inactivate virus particles by ultraviolet light (UV), the virus solutions were placed in 60-mm petri dishes and positioned close to the UV lamp to ensure the UV exposure at 0.4 J/m^2^. An un-treated sample served as control. Successful UV-inactivation was determined by Standard Plaque Titration Assay.

### Viral infections and Standard Plaque Titration Assay

For infection, cells were washed with PBS and incubated with IAV at the indicated multiplicities of infection (MOI) as previously described^60^. In case of infection with H1N1 viruses the medium was supplemented with 2 μg/ml trypsin (Invitrogen). To assess progeny virus titres, supernatants of infected cells were collected at indicated times. The number of infective particles were determined by Standard Plaque Titration Assay as described earlier^61^.

### Stimulation with PGE2 and CAY10404

A549 cells were pre-incubated with 5 μg/ml PGE2 (Sigma-Aldrich), diluted in ethanol, or 5 μM COX-2 inhibitor CAY10404 (Cayman Chemicals), diluted in DMSO or the respective solvent as control for 1 h. Subsequently, cells were infected with IAV in addition to the treatment with PGE2, CAY10404 or the respective solvent for 24 h. Viral titres were determined by Standard Plaque Titration Assay.

In time-of-addition kinetics A549 were either pre-incubated with 5 μM CAY10404, or 5 μg/ml PGE2 or the respective solvent for 1 h prior infection or left untreated. Subsequently, cells were infected with FPV (MOI 0.01). Substances were added at the indicated times of infection and viral titres were determined by Standard Plaque Titration Assay 10 h p.i.

### Western Blotting and antibodies

Protein lysates were generated by using radio-immunoprecipitation assay (RIPA) buffer as previously described^60^ and analysed by sodium dodecyl sulphate polyacrylamide gel electrophoresis (SDS-PAGE) and Western Blot (WB) analysis. For the detection of proteins anti-COX-2 rabbit polyclonal antibody from Cell Signaling Technology, anti-M1 mouse monoclonal antibody from Biorad, anti-NS1 mouse monoclonal antibody (clone NS1-23-1, developed at the Institute of Molecular Virology, Muenster, Germany), anti-α-Tubulin mouse monoclonal antibody and anti-flag M2 mouse monoclonal antibody from Sigma Aldrich, anti-COX-2 goat polyclonal antibody, anti-ERK2 (C-14) rabbit polyclonal antibody, anti-PB1 (vK-20) goat polyclonal antibody and anti-TTP (H-120) rabbit polyclonal antibody all from Santa Cruz Biotechnology, anti-phospho-STAT1 Tyr^701^ (clone 14) mouse monoclonal antibody from BD Bioscience as well as anti-RIG-I mouse monoclonal antibody from Alexis Biochemicals (Enzo Life Sciences) were used.

### Isolation and de-phosphorylation of RNA

Total RNA from infected or stimulated A549 cells was extracted using RNeasy Mini Kit (QIAGEN). RNA isolated from un-infected or not stimulated cells served as control.

For the de-phosphorylation of RNA, Antarctic Phosphatase (New England Biolabs) was used according to the manufacturer’s protocol.

### Plasmids, siRNA and transient transfection

The plasmids *pCMV-XL6-COX-2* (OriGene Technologies, USA), *pCAGGS-Rig-I*^47^, and the murine *p3XFLAG-CMV-7.1-TTPcds*^62^ were used for overexpression of the respective proteins in A549 cells. Corresponding empty vectors served as controls.

For knock-down, human RIG-I siRNA as well as negative control siRNA were purchased from QIAGEN, and human TTP siRNA was purchased from Santa Cruz Biotechnology. All siRNAs were used at 10 μM.

A549 cells were transfected with Lipofectamine^®^ 2000 (Invitrogen) transfection reagent according to the manufacturer’s protocol.

### Quantitative real-time PCR

Prior to quantitative real-time PCR (qRT-PCR) the mRNA had to be reverse transcribed with 0.5 μg oligo(dT) primer, 10 mM dNTPs, 5x reaction buffer and 200 U Revert Aid H Minus M-MuLV (Thermo Scientific) for 1 h at 42 °C in a total volume of 20 μl. Reverse transcriptase was denatured via heat-inactivation for 10 min at 70 °C. According to manufacturer’s instruction Brilliant III SYBR^®^ Green QPCR Master Mix (Agilent Technologies) was used for qRT-PCR. The gene expression of the housekeeping gene *gapdh* was determined as internal standard^63^.

Used primers were: GAPDH forward, 5′-GCA AAT TTC CAT GGC ACC GT-3′, GAPDH reverse, 5′-GCC CCA CTT GAT TTT GGA GG-3′, COX-2 forward, 5′-TTC AAA TGA GAT TGT GGG AAA ATT GCT-3′, COX-2 reverse, 5′-AGA TCA TCT CTG CCT GAG TAT CTT-3′, TTP forward 5′-CGC TAC AAG ACT GAG CTAT-3′ and TTP reverse 5′-GAG GTA GAA CTT GTG ACA GA-3′.

### mRNA stability assay

COX-2 mRNA stability was analysed by inhibition of new mRNA synthesis via actinomycin D (ActD, Santa Cruz Biotechnology). A549 cells were infected with 5 MOI of IAV, or transfected with 1 μg RNA, isolated from infected A549 cells or mock-infected A549 cells, or stimulated with 1 μg lipopolysaccharide (LPS, InvivoGen) for 4 hours before 3 μg ActD was added. Cells were subsequently harvested after 0, 1 and 2 h of ActD treatment. RNA was isolated, reverse transcribed and quantified by qRT-PCR, as described above.

### Statistical Analysis

All experiments were performed in at least three independent settings. Representative data are shown for Western Blot analysis. Other data are expressed as the mean ± s.d. of the independent experiments. Statistical significance was determined by using GraphPad Prism software version 6. Used statistical tests are described in figure legends.

## Additional Information

**How to cite this article**: Dudek, S. E. *et al*. Influenza A viruses suppress cyclooxygenase-2 expression by affecting its mRNA stability. *Sci. Rep.* **6**, 27275; doi: 10.1038/srep27275 (2016).

## Supplementary Material

Supplementary Information
