# Supplementary material for: Influenza A viruses suppress cyclooxygenase-2 expression by affecting its mRNA stability
Source: Sci Rep. 2016 Jun 6;6:27275. doi: 10.1038/srep27275 (PMC4893666; doi:10.1038/srep27275)
Supplement: Supplementary Information [file srep27275-s1.doc]

**Influenza A viruses suppress cyclooxygenase-2 expression by affecting its mRNA stability**

Sabine Eva Dudek^1^, Katja Nitzsche^1^, Stephan Ludwig and Christina Ehrhardt*

Institute of Molecular Virology (IMV), Centre for Molecular Biology of Inflammation (ZMBE), Westfaelische Wilhelms-University Muenster, Von-Esmarch-Str. 56, D-48149 Muenster, Germany

^1^ These authors contributed equally to this work.

*Corresponding author

PD Dr. Christina Ehrhardt

Phone: +49 251 8353010

Fax: +49 251 8357793

e-mail: ehrhardc@uni-muenster.de

**Supplementary Material**

*Primary cells*

Primary human bronchial epithelial cells (HBEpC) were obtained from Promocell and cultivated in airway epithelial growth medium supplemented with 4 µl/ml bovine pituitary extract, 10 ng/ml epidermal growth factor, 5 µg/ml insulin, 0.5 mg/ml hydrocortisone, 0.5 mg/ml epinephrine, 6.7 ng/ml triiodo-L-thyronine, 10 µg/ml transferrin and 0.1 ng/ml retinoic acid at 37 °C in a humidified 5% CO_2_ atmosphere. All experiments were performed at cell population doubling number 11.

*Viruses*

The NS1-deletion mutant virus A/Puerto-Rico/8/34/dNS1 (H1N1, ΔNS1) was propagated and passaged in MDCK cells.

*siRNA and transfection of siRNA*

IFNAR2 siRNA and control siRNA were purchased from QIAGEN. A549 cells were transfected with 10 µM of siRNA with Lipofectamine® 2000 (Invitrogen) according to manufacturer’s protocol. Experiments were performed 48 h post transfection.

*Antibodies and neutralisation experiments*

Anti-Phospho-STAT1 Y701 (clone14) mouse monoclonal antibody was obtained from BD Bioscience. Anti-IFNβ goat polyclonal neutralising antibody was purchased from Abcam. For neutralisation experiments A549 cells were simultaneously stimulated with different amounts of IFNβ (pbl Assay Science) and with or without the neutralising antibody in a concentration of 4 μg/ml medium for 24 h.

**Supplementary Figures**

**
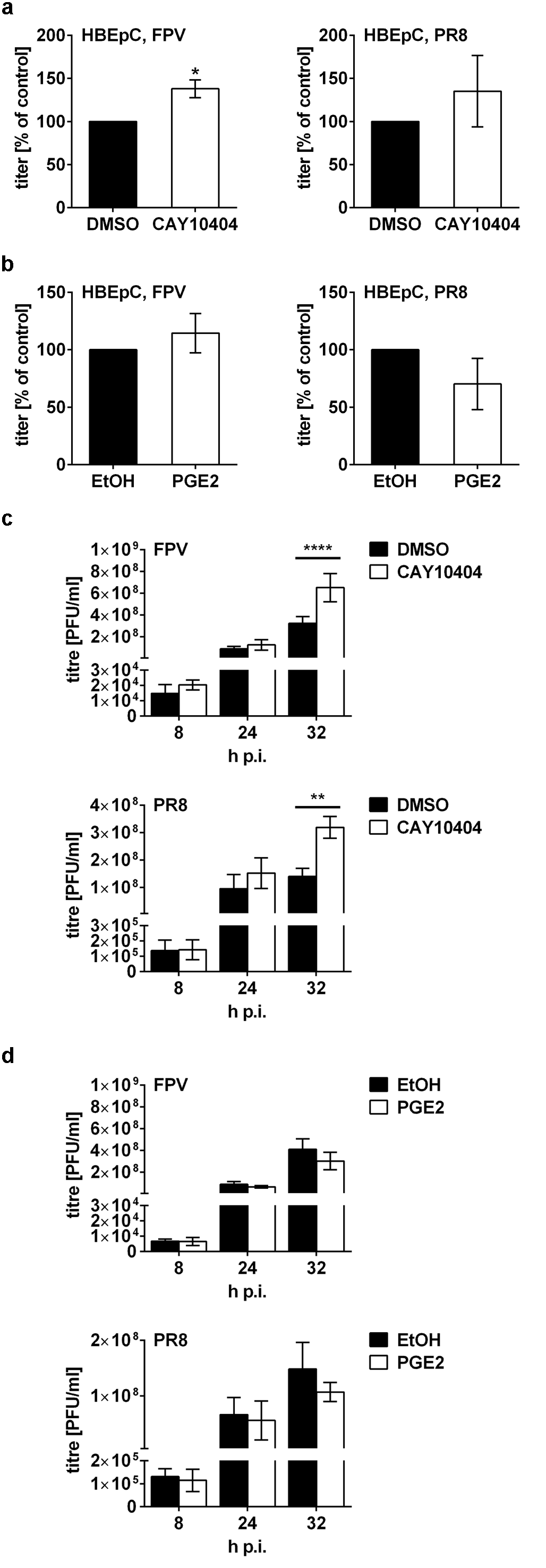
**

**Supplementary Figure S1: Influence of COX-2 and its product PGE2 on IAV replication.** (a, b) Primary human bronchial epithelial cells (HBEpC) were pre-incubated with 5 µM of COX-2 inhibitor CAY10404 or the solvent DMSO (a), or with 5 µg/ml PGE2 or the solvent ethanol (EtOH) (b) for 1h. Subsequently, cells were infected with the IAV subtypes H7N7 (FPV) or H1N1 (PR8) at an MOI of 1 in addition to the treatment with CAY10404 (a), PGE2 (b) or the respective solvent for 24 h. Viral titres were determined by Standard Plaque Titration Assay. Results are depicted as mean (± s.d.) in % of solvent control (n=3). Statistical significance was determined by using unpaired t-test with Welch’s correction. *, P = 0.0235. (c, d) A549 cells were pre-incubated with 5 µM of COX-2 inhibitor CAY10404 (c), or with 5 µg/ml PGE2 (d) or the respective solvent for 1h. Subsequently, cells were infected with the IAV subtypes H7N7 (FPV) at an MOI of 0.001 or H1N1 (PR8) at an MOI of 0.01 in addition to the treatment with CAY10404 (c), PGE2 (d) or the respective solvent for times indicated. Viral titres were determined by Standard Plaque Titration Assay. Results are depicted as mean (± s.d.) of absolute titres as plaque forming units per ml (PFU/ml) (FPV, n=3; PR8, n=2). Statistical significance was determined by using two-way ANOVA followed by Sidak’s test. **, P = 0.0088, ****, P < 0.0001.


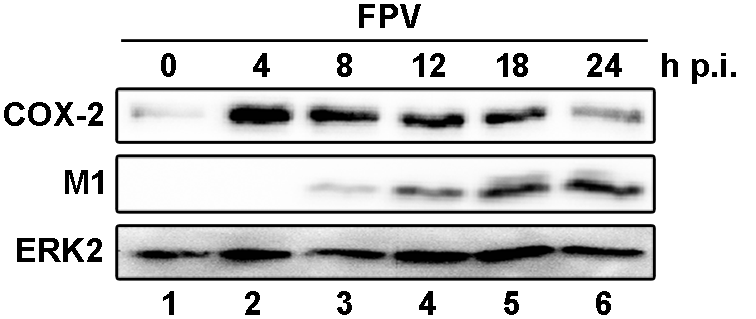


**Supplementary Figure S2: COX-2 expression is reduced during on-going IAV infection in primary epithelial cells.** HBEpC were infected with 1 MOI of the IAV subtype H7N7 (FPV) for the times indicated. Total cellular protein extracts were analysed by Western Blot (WB). Shown is one representative blot of n=3 independent experiments.


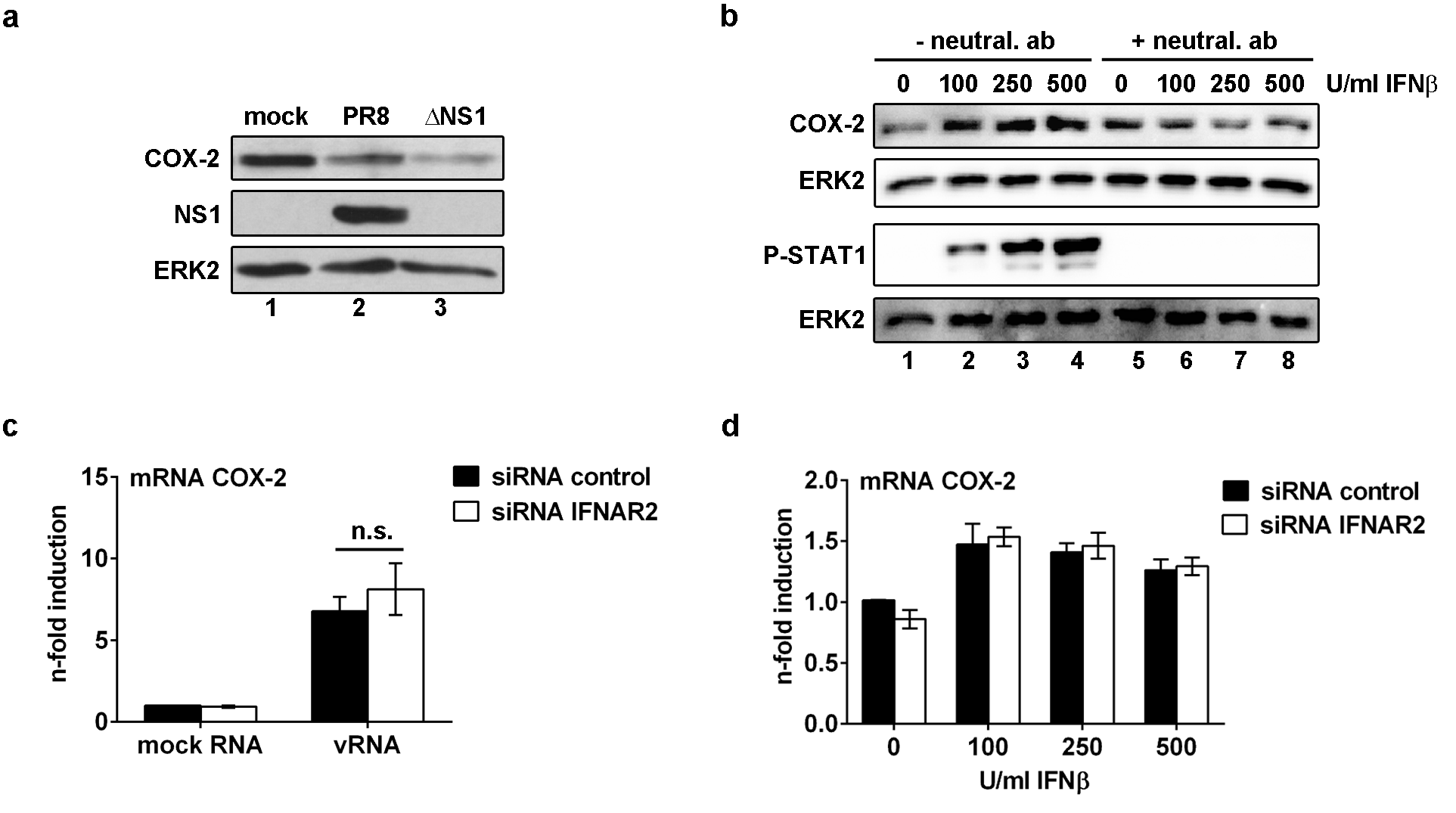


**Supplementary Figure S3: COX-2 reduction is not dependent on type I IFN-mediated signalling.** (a) A549 cells were mock-treated or infected with 1 MOI of PR8 or the NS1 deletion mutant (ΔNS1) virus for 24 h. Total cellular protein extracts were analysed by WB. Shown is one representative result of n=2 independent experiments. (b) A549 cells were stimulated with indicated concentrations of IFNβ in presence (+ neutral. ab) or absence (- neutral. ab) of a neutralising anti-IFNβ antibody for 24 h and analysed by WB. P-STAT1 (Y709) served as control of IFNβ neutralisation. Shown is one representative result of n=2 independent experiments. (c and d) A549 cells were transfected with an siRNA specific for the IFNα/β receptor 2 (IFNAR2) or a control siRNA (control) for 48 h. (c) Cells were transfected with 1 μg RNA, extracted from IAV-infected A549 cells (vRNA) or mock-infected A549 cells (mock RNA) for 4 h, (d) or stimulated with IFNβ at the indicated amounts for 24 h. (c and d) Total RNA was isolated, reverse transcribed and analysed by qRT-PCR. Expressional changes of COX-2 mRNA were normalised to (c) mock RNA control, or (d) unstimulated control, respectively. Results are depicted as mean n-fold (± s.d.) of n=3 independent experiments. Statistical significance was determined using two-way ANOVA followed by Sidak’s test showing no significant differences (n.s.).


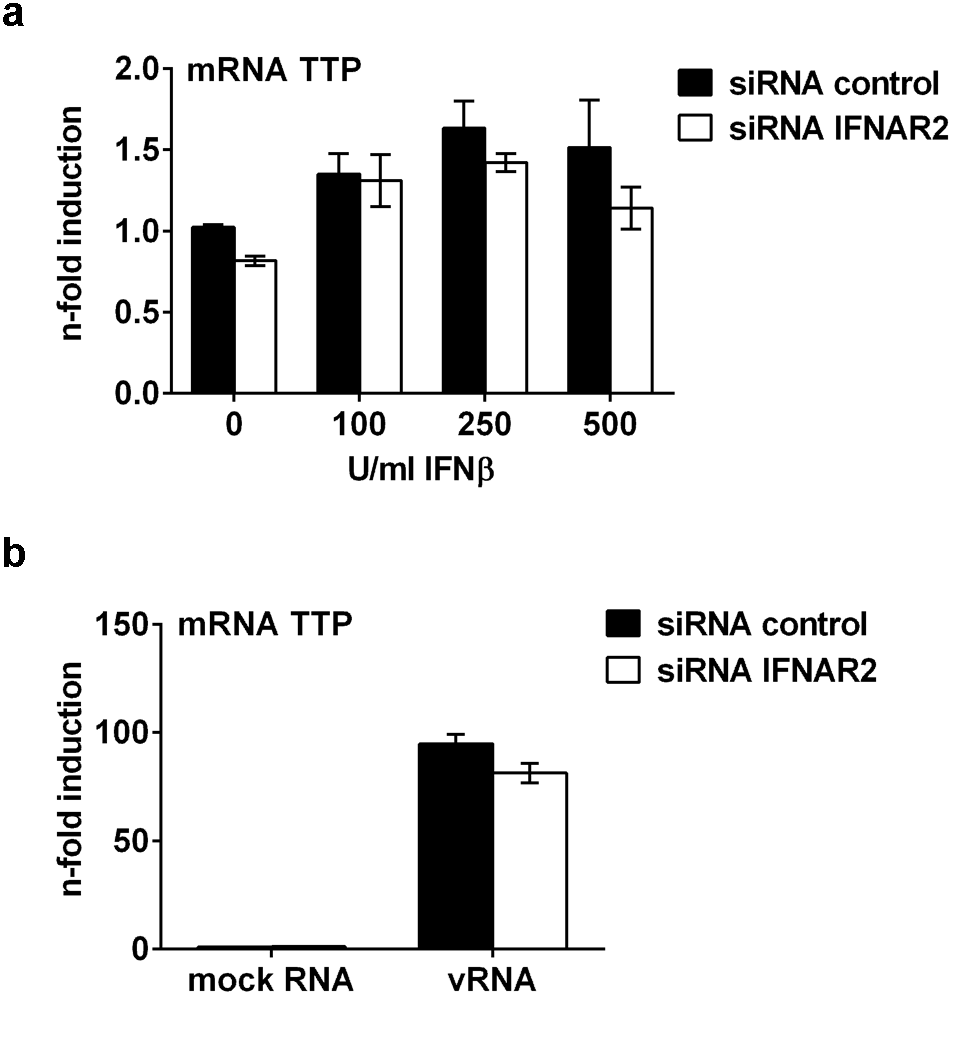


**Supplementary Figure S4: Induction of TTP mRNA is independent of type I IFN signalling.** (a and b) A549 cells were transfected with an siRNA specific for the IFNα/β receptor 2 (IFNAR2) or a control siRNA (control) for 48 h. (a) Subsequently, cells were stimulated with IFNβ at the indicated amounts for 24 h, (b) or transfected with 1 μg RNA, extracted from IAV-infected A549 cells (vRNA) or mock-infected A549 cells (mock RNA) for 4 h. (a and b) Total RNA was isolated, reverse transcribed and analysed by qRT-PCR. Expressional changes of TTP mRNA were normalised to (a) unstimulated control or (b) mock RNA control, respectively. Results are depicted as mean n-fold (± s.d.) of n=3 independent experiments. Statistical significance was determined using two-way ANOVA followed by Sidak’s test showing no significant differences.
